# The Effectiveness of Synbiotic Preparation Containing *Lactobacillus* and *Bifidobacterium* Probiotic Strains and Short Chain Fructooligosaccharides in Patients with Diarrhea Predominant Irritable Bowel Syndrome—A Randomized Double-Blind, Placebo-Controlled Study

**DOI:** 10.3390/nu12071999

**Published:** 2020-07-05

**Authors:** Barbara Skrzydło-Radomańska, Beata Prozorow-Król, Halina Cichoż-Lach, Emilia Majsiak, Joanna B. Bierła, Wojciech Kosikowski, Mariusz Szczerbiński, Jesper Gantzel, Bożena Cukrowska

**Affiliations:** 1 Department of Gastroenterology, Medical University of Lublin, Jaczewskiego 8, 20-950 Lublin, Poland; barbara.radomanska@gmail.com (B.S.-R.); prozorow1@wp.pl (B.P.-K.); lach.halina@wp.pl (H.C.-L.); szczerbin@mp.pl (M.S.); 2 Faculty of Medicine, Cardinal Stefan Wyszynski University, Wóycickiego 1/3, 01-938 Warszaw, Poland; e.majsiak@interia.pl; 3 Department of Pathology, The Children Memorial Health Institute, Aleja Dzieci Polskich 20, 04-730 Warsaw, Poland; j.bierla@ipczd.pl; 4 Out-Patient Clinic, Konopnica 96L, 21-030 Motycz, Poland; wz.kosikowski@gmail.com; 5 Biocare Copenhagen, Ole Maaloes Vej 3, DK-2200 Copenhagen, Denmark; jng@zel.dk

**Keywords:** irritable bowel syndrome, synbiotics, probiotics, prebiotics, short chain fructooligosaccharides, *Lactobacillus*, *Bifidobacterium*

## Abstract

The purpose of the randomized double-blind placebo-controlled trial was to assess the effectiveness of synbiotic preparation containing probiotic *Lactobacillus rhamnosus* FloraActive™ 19070-2, *Lactobacillus acidophilus* DSMZ 32418, *Bifidobacterium lactis* DSMZ 32269, *Bifidobacterium longum* DSMZ 32946, *Bifidobacterium bifidum* DSMZ 32403 and fructooligosaccharides in adult patients with diarrhea-dominant IBS (IBS-D). The study included eighty patients with moderate and severe IBS-D who were randomized to receive synbiotics or placebo for eight weeks. Finally, a total of sixty-eight patients finished the study. The primary endpoints included the assessment of the symptoms’ severity with IBS symptom severity scale (IBS-SSS), an improvement of IBS global symptoms with Global Improvement Scale (IBS-GIS) and adequate relief of symptoms after four and eight weeks of therapy. Secondary endpoints, which were collected by telephone interviewers three times a week included the assessment of individual IBS symptoms and adverse events. Synbiotic treatment in comparison to placebo significantly improved IBS-GIS (*p* = 0.043), and IBS-SSS score inducing a decrease in the total IBS-SSS (*p* = 0.042) and in domain-specific scores related to flatulence (*p* = 0.028) and bowel habit (*p* = 0.028) after four and eight weeks. Patients treated with synbiotics reported in weekly observations a significant amelioration in a feeling of incomplete bowel movements, flatulence, pain, stool pressure and diarrheal stools compared to those receiving placebo. There were no differences in adverse events between both groups. Concluding, the multi-strain synbiotic preparation was associated with a significant improvement in symptoms in IBS-D patients and was well-tolerated. These results suggest that the use of synbiotics offers a benefit for IBS-D patients. [Clinicaltrials.gov NCT04206410 registered 20 December 2019].

## 1. Introduction

Irritable bowel syndrome (IBS) is a chronic functional gastrointestinal disorder characterized by abdominal pain and altered bowel habits without any structural abnormalities [1]. IBS affects approximately 10% to 25% of the adult population worldwide [2]. Patients experience abdominal pain and altered bowel habit, with either predominantly diarrhea (IBS-D), constipation (IBS-C), or both (IBS-M). There are no definitive biomarkers of the disease, so IBS is diagnosed clinically according to Rome criteria [3].

Despite extensive research, the pathogenesis of IBS has not yet been clearly elucidated yet. Recent studies have shown that disturbed gut microbiota may promote the development and maintenance of IBS [4,5,6]. Alterations in the intestinal microbiota (dysbiosis) could contribute to IBS by increasing gut permeability, activating the mucosal inflammatory immune response, increasing visceral sensitivity, altering intestinal motility [7,8,9]. In addition, the onset of IBS following infective gastroenteritis and the involvement of small bowel bacterial overgrowth (SIBO) suggest that gut microbes play a role in IBS pathogenesis [10]. IBS can be also seen as a “stress disease”, and there is evidence that the microbiome-gut-brain axis is disturbed in IBS.

Significant changes in the microbial communities of healthy controls vs. IBS patients have been reported in several studies [11,12,13]. Based on the analysis of fecal samples, decreased proportions of the genera *Bifidobacterium* and *Lactobacillus* and increased ratios of *Firmicutes:Bacteroidetes* at the phylum level were observed. Molecular analyses of mucosal brush samples or luminal aspirates have indicated a decreased diversity in small-bowel microbiota of patients with IBS, with an abundance of gram-negative organisms. Recently, a reduction in butyrate- and methane-producing bacteria in patients with IBS-D and IBS-M was found [14].

These findings promoted the research on probiotics and synbiotics for the treatment of IBS. Probiotics are live microorganisms which, when administered at the right dose, have a positive effect on human health [15]. Synbiotics are a mixture of probiotics and prebiotics (most often oligosaccharides selectively utilized by bacteria), which act synergistically to promote the growth and survival of beneficial microorganisms in the gut [16].

The currently published systemic reviews and meta-analyses of randomized clinical trials have indicated that probiotics have beneficial clinical effects and can help to reduce global and specific IBS symptoms significantly [17,18,19,20]. In contrast, little evidence for the use of synbiotics in IBS has been demonstrated [18,19]. Unlike probiotics, whose clinical effects have been evaluated in numerous studies (59 randomized double-blind clinical trials, enrolling 6761 IBS patients, were included in the 2020 meta-analysis [17]), there are few clinical trials showing the effects of synbiotics. Only two studies assessing the importance of synbiotics in the treatment of IBS were included in the meta-analysis published by Ford et al. in 2018 [15]. One of the studies was single-blinded [21], and the other evaluated the effects of synbiotics as yogurt supplements [22]. The latest meta-analysis published in 2020 by Asha et al. [19] included other three randomized double-blind placebo controlled clinical trials in which adult patients with IBS were given synbiotic preparations. However, the results of clinical trials with the use of synbiotics are not conclusive, and authors of meta-analyses emphasize that further research with the use of synbiotics is necessary.

Therefore, the purpose of the current clinical trial is to assess the effectiveness of synbiotic preparation containing probiotic *Lactobacillus* and *Bifidobacterium* strains and short chain fructooligosaccharides (scFOS) in patients with IBS-D.

## 2. Materials and Methods

### 2.1. The Study Design

The study was randomized double-blind placebo controlled equal location ratio parallel group performed at outpatient clinics between August 2018 and September 2019. The study was approved by Bioethical Committee of the Children’s Memorial Health Institute (decision number 6/KBE/2018). All participants were informed about the objectives, methodology and purpose of the study, and those who agreed to participate were required to provide written consent prior to entry. The study was conducted in accordance with the ethical principles set out in the Declaration of Helsinki Guideline on Good Clinical Practice. [Clinicaltrials.gov NCT04206410 registered 20 December 2019].

### 2.2. Patients

The study included female and male patients aged 18–60 years with IBS-D diagnosed according to Rome III criteria (recurrent abdominal pain or discomfort defined as an uncomfortable sensation not described as pain at least three days a month in the past three months, associated with improvement with defecation; IBS onset associated with a change in frequency and form of stool) [23]. Patients classified as IBS-D had at least one stool of type 5, 6 or 7 in Bristol stool score over at least 2 days a week [24]. The severity of IBS was determined by the IBS Severity Scoring System (IBS-SSS) [25]. The patients who presented moderate (>175 points) and severe type of IBS (>300 points) were enrolled in the study.

The exclusion criteria included: the use of probiotics (both recommended by a physician and self-taken) and treatment with antibiotics within last three months; a concurrent severe illness (malignancies, uncontrolled hypertension and diabetes mellitus, hepatic, renal or cardiac dysfunctions, serious neurological disorders, psychosis, respiratory disorders such as asthma, chronic obstructive pulmonary disease, hyper- or hypothyroidism); chronic bowel disorders other than IBS, including inflammatory bowel diseases, gastroenteritis, stomach and duodenal ulcers, celiac diseases; pregnancy or lactation; diagnosed lactose intolerance; the use of motility drugs or dietary fiber supplements within 2 weeks before study start; plan to have surgery during the time of the study; a history of alcohol or drug abuse, taking anti-coagulant medications, participation in another clinical trial within last three months. Patients who received antibiotics during the study also were excluded. Patients were allowed to take low-dose anti-depressants (up to 25 mg per day of amitriptyline, nortriptyline or selective serotonin inhibitor). They could also take spasmolytic drugs on an ad hoc basis.

### 2.3. The Synbiotic Preparation

The synbiotic preparation in the form of sachets contained multi-strain probiotic mixture of three *Bifidobacterium* and two *Lactobacillus* species (Table 1). The total number of colony-forming units (CFU) per sachet was five billion. The composition of probiotic bacteria resulted from an earlier preliminary study in which positive effects in IBD patients were observed [26]. Patients obtained a mixture of four strains (*Bifidobacterium lactis, Bifidobacterium longum, Bifidobacterium bifidum* and *Lactobacillus acidophilus*) that were used in the present study. Additionally, the probiotic mixture was enriched with *Lactobacillus rhamnosus* FloraActive 19,070 strain, which when administered to babies with infant colic significantly reduced the time of crying and anxiety compared to the control group [27]. We decided on a dose of 10 billion/day, taking into account the results of the meta-analysis assessing the optimal dose of probiotics in IBS, which showed that the dosage of 10^9^–10^10^ CFU/day may be a reference range [28].

The synbiotic preparation also contained prebiotic scFOS from Actilight^®^ (Beghin Meiji, Marckolsheim, France) in a dose of 947 mg in each sachet. scFOS was obtained from sugar beet by a bio-enzymatic reaction and contained 44% of glucose linked 2 units of fructose (GF2), 46% GF3, and 10% GF4. The molecular weight of scFOS was 603 g/mol, and purity of scFOS was at least 95% of dry substance: The placebo sachets contained 978 mg of maltodextrin comparable in color, texture and taste to the synbiotic mixture. The synbiotic preparation and placebo were supplied by Biocare Copenhagen, Denmark, which blinded the products. Samples were marked on the collective packaging (14 sachets in one packaging) and on individual sachets as a product A and B. The packaging looked identical and contained the title of the study, the approval number of the Bioethical Committee, and expiry date. Two batches of each product were produced and had a 2-year shelf life. The products were refrigerated below 6 °C until they were distributed to the clinics, where they were stored at room temperature. Products were stored in the refrigerator for no longer than 8 months, while it was issued to the physicians every 3–4 months as needed. The quality of products was monitored by Biocare Copenhagen, Denmark, and the CFU number was stable throughout the shelf-life of the synbiotic preparation.

### 2.4. Scheme of the Study

**Figure 1:**
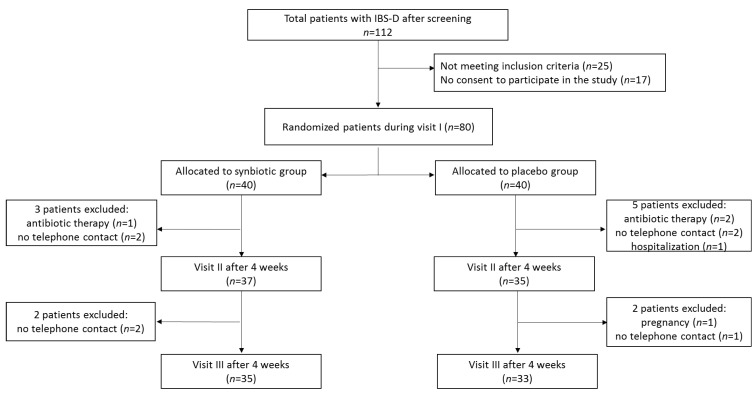
The flowchart of study protocol.

A 2-week screening period was used to establish the presence and persistence of clinical inclusion criteria and to train patients how to collect data. During screening visit, patients underwent physical examination and severity of IBS was determined using IBS severity scale (IBS-SSS). They were also instructed to restrain from consuming foods and dietary supplements containing any probiotics according to the clinical study requirements. Out of 328 patients with IBS 112 fulfilled inclusion criteria for IBS severity (IBS-SSS score >175), but 25 of them did not meet other inclusion criteria, and 17 did not agree to participate in the study (Figure 1). Finally, 80 patients meeting all inclusion criteria signed informed consent form.

During the first visit (visit I), all eligible patients were assessed by a physician with the use of IBS-SSS questionnaire and were allocated according to a computer-generated blocked list with a block size of 2 to the synbiotic (patients receiving a synbiotic preparation) or placebo group. The block size was not disclosed to the investigators, and the allocation was blinded to both patients and investigators. Patients received one sachet with the synbiotic preparation or an identical appearing and tasting placebo (maltodextrin) 2 times a day over an 8-week period. The contents of sachets were ingested after being dissolved in 100 mL of water. Patients reported to a physician every 4 weeks (visit II and visit III) in order to receive a synbiotic preparation or placebo for the next 4 weeks (visit II) and to be clinically assessed.

After including patients in the trial, telephone interviewers called patients 3 times per week and collected the following information over the phone: the numbers of bowel movements per day, the type of stools and IBS symptoms (abdominal pain, flatulence, urgency for bowel movement, abdominal distension after bowel movement). In addition, telephone interviewers collected information regarding adverse effects.

### 2.5. Endpoint Definitions

The prospective defined primary efficacy variables were: (1) the assessment of the severity of IBS symptoms using IBS-SSS, (2) the assessment of improvement or worsening of IBS global symptoms using Global Improvement Scale (IBS-GIS) and (3) the assessment of adequate relief of IBS symptoms (IBS-AR).

IBS-SSS was a 5-question survey that focused on: (1) the severity of abdominal pain (IBS-SSS1), (2) the frequency of abdominal pain over the last 10 days (IBS-SSS2), (3) the severity of abdominal flatulence (IBS-SSS3), (4) dissatisfaction with bowel habit (IBS-SSS4) and (5) interference with quality of life over the past 10 days (IBS-SSS5) [25]. Subjects responded to each question on a 100-point visual analogue scale. Each of the five questions generated a maximum score of 100 points, and total scores could range from 0–500 with higher scores indicating severe symptoms. A decrease of 50 points (50%) compared with baseline (Visit I) was associated with a clinically meaningful improvement.

IBS-GIS assessed IBS symptoms using a patient-defined 7-point Likert scale ranging from symptoms substantially worse (1 point) to substantially improved (7 points) [29]. Patients answered the question “Have you felt any change in the severity of your symptoms over the past 7 days compared to how you felt before the medicine was taken?” The answers were recorded based on the 7-point scale: 1 point—“I feel that the symptoms have worsened significantly”; 2 points—“I feel that the symptoms have moderately worsened”; 3 points—“I feel that the symptoms have slightly worsened”; 4 points—“I feel no change”; 5 points—“I feel a slight improvement”; 6 points—“I feel moderate improvement”; 7 points—“I feel significant improvement”. IBS-GIS score indicated an improvement if score was >4, worsening if it was <4 and no change if it was 4.

IBS-Adequate Relief (IBS-AR) was a dichotomous single item that asked participants “Over the past week have you had adequate relief of your IBS symptoms?” The answer was YES or NO.

Secondary efficacy variables included: (1) the type of stools assessed using Bristol stool scale, (2) the frequency of bowel movements per day, (3) the severity of pain, (4) the severity of flatulence, (5) stool pressure, (6) the feeling of incomplete evacuation of stool and (7) adverse events. All information was collected by telephone interviewers 3 times a week before interventions and for 8 weeks of interventions. The type of stools was assessed using the Bristol Stool Scale that classified feces into seven groups: types 1–2 indicated constipation, types 3–4 were “normal” stools and types 5–7 indicated diarrhea. IBS symptoms except for a feeling of incomplete bowel movement were assessed using a patient-defined 5-point Likert scale: 0 points indicating no symptoms and 1–4 indicating the severity of symptoms with higher scores indicating worse symptoms. A feeling of incomplete bowel movement was assessed using a 2-point scale: 0—no such feeling and 1—there is an incomplete bowel movement. Adverse events were evaluated on the scale from 0 (no) to 1 (yes). To reduce the dimension of the dataset from telephone interview level (day), the data were transformed to weekly level observations, and primary scores of Bristol stool scale and 5-point Likert scale were transformed to 3-point “novel scores” presented in Table 2. Finally, for each week, the weekly level of the type of defecation, the severity of pain, flatulence, pressure, feeling of incomplete defecation and side effects was calculated as the most common (dominant) value, and the number and percentage of patients with this value was calculated.

### 2.6. Statistical Analyses

Data were analyzed using Stata Program version 12.1 (StataCorp LLC, College Station, Texas, USA). Differences in sex and number of patients with an improvement or worsening of IBS symptoms between the synbiotic and the placebo groups were evaluated with the use of a Fisher exact test. Respective differences in age and physical development parameters distribution were examined using two-sample Wilcoxon rank-sum test. The analyses of continuous variables across subsequent visits or weekly telephone interviews were based on RM-ANOVA after testing the normality assumption using a Shapiro–Wilk test. The analyses of nominal variable measured on the Bristol stool scale and Likert scale were done by two-sided paired or unpaired t-tests after checking for equality of variances and normality assumption using a Shapiro–Wilk test. In the case normality assumption did not hold, two-sample Wilcoxon paired or unpaired signed-rank test was used. Weekly changes in symptoms (measured as no or weak symptoms (0), moderate symptoms (1), severe symptoms (2)), type of stool (measured as constipation (0), normal stools (1), diarrhea (2)), the feeling of incomplete bowel movement and adverse events (measured as 0 (none), 1 (present)) were analyzed with the use of a Fisher exact test (naïve approach ignoring the weeks) and Cochran–Mantel–Haenszel test (where the third variable indicating the week was introduced).

The results regarding IBS-SSS are presented as arithmetic means ± standard deviation (SD) of IBS-SSS scores and changes from baseline and percentages of patients with an improvement (a decrease of scores by 50 points or more)/no improvement during intervention. The results for IBS-GIS are presented as arithmetic means ± SD and for IBS-GIS and IBS-AR (after data transformation) as percentages of patients with an improvement/no improvement of symptom scores comparing with baseline. The results for IBS symptoms and adverse events obtained via telephone interviews are shown as arithmetic means ±SD of changes from baseline and as percentages of patients with an improvement/no improvement defined by the scale transformation (Table 2).

## 3. Results

### 3.1. Subjects

A total of 80 patients were randomized to receive the synbiotic preparation or placebo (Figure 1). After the 4-week treatment (visit II), three patients from the synbiotic group and five patients from the placebo group were excluded because of antibiotic treatment (*n* = 1 in the synbiotic groups and *n* = 2 in the placebo group), no telephone contact (*n* = 2 in the synbiotic group and *n* = 2 in the placebo group) and one urgent hospitalization due to heart failure in the placebo group. In the next 4 weeks, two patients from each group were excluded from the study. Finally, a total of 68 patients (35 receiving the synbiotic preparation and 33 placebo) finished the study. The patient characteristics are presented in Table 3. Patients were predominantly female in both groups and constituted 71.4% and 72.7%, respectively, in the synbiotic and the placebo groups. There were no statistically significant differences between the synbiotic and the placebo groups in gender, age, physical development and IBS severity.

### 3.2. Study Primary Endpoints

The primary outcomes included the reduction of IBS-symptoms determined using IBS-SSS, IBS-GIS and IBS-AR questionnaires after treatment. The mean values of the total IBS-SSS before the intervention were similar in both groups: 318.1 ± 63.6 and 325.5 ± 49.9 in the synbiotic and placebo groups, respectively (Table 4). The statistically significant decrease in IBS severity between the baseline and visit II (after 4-week treatment) and visit III (after 8-week treatment) was found in both groups (*p* < 0.001). There were no differences in the total IBS-SSS score between both groups after 4-week intervention, but after 8 weeks, a statistically significant reduction of a score (*p* = 0.042) related to symptom amelioration was found in the synbiotic group (change from baseline −169.6 ± 88.7 points) in comparison to the placebo group (−141.8 ± 95.4) with domain-specific scores related to flatulence (IBS-SSS3). Compared to the placebo group, patients receiving synbiotics experienced significantly less flatulence not only after 8 weeks of treatment (*p* = 0.028) but already after 4 weeks (*p* = 0.039). There were no differences between both groups in other symptoms or quality of life.

**Figure 2:**
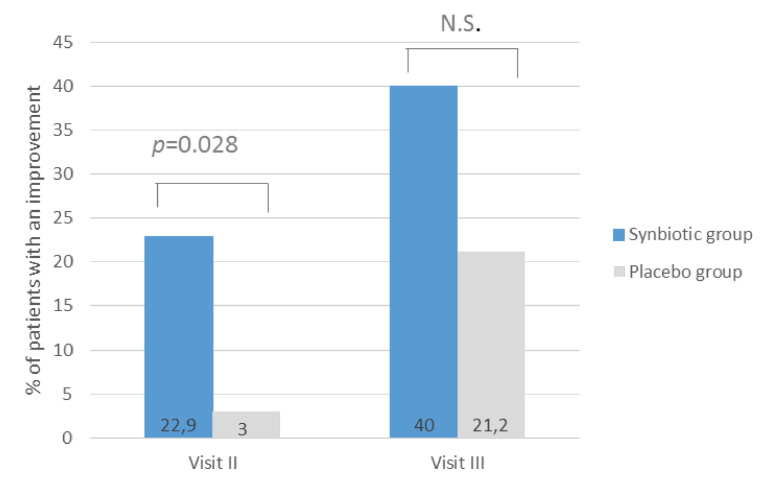
The percentage of patients reported an improvement in dissatisfaction with bowel habits assessed by IBS-SSS scale after synbiotic intervention lasting 4 weeks (visit II) and 8-weeks (visit III). An improvement was defined as a 50-point decrease compared to baseline. N.S. = no significance (*p* > 0.05).

When we assumed that significant responders to intervention were patients defined by 50-point (50%) reduction compared to baseline, the question regarding the assessment of dissatisfaction with bowel habits (IBS-SSS4) presented significant differences between studied groups (Figure 2). After 4 weeks treatment (Visit II), the percentage of patients with a 50-point decrease was statistically significantly higher (*p* = 0.028) in the synbiotic group compared to the placebo group, which were 22.9% and 3.0%, respectively. After 8 weeks, the percentage of patients showing improvement in the dissatisfaction of bowel habit assessment increased in the synbiotic group to 40% and was almost 2 times higher than in the placebo group but did not reach statistical significance. Other symptoms assessed on the IBS-SSS scale did not show statistical significance between the examined groups (Table A1).

**Figure 3:**
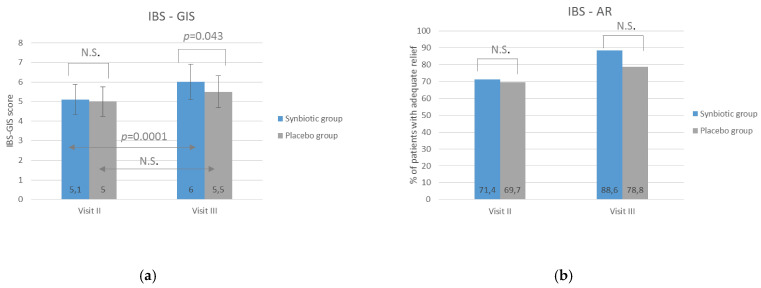
The impact of synbiotic treatment on the global improvement of IBS-symptoms (IBS-GIS) (**a**) and adequate relief (IBS-AR) (**b**). IBS-GIS and IBS-AR were evaluated after 4 weeks (visit II) and 8 weeks (visit III) of treatment. The results are presented as means of IBS-GIS score or the percentage of patients with adequate relief. A statistically significant improvement of IBS-GIS score between visits II and III was observed only in the synbiotic group, and a significantly amelioration in the synbiotic group compared with the placebo group was found after 8-week lasting treatment (visit III). No statistically significant differences were found in IBS-AR. N.S. = no significance (*p* > 0.05).

The results suggesting the positive impact of the synbiotic intervention on the severity of IBS symptoms assessed by the IBS-SSS scale were confirmed by the IBS-GIS scale related to the global improvement of symptoms compared to the baseline. After a 8-week lasting intervention (Visit III), patients from the synbiotic group statistically significantly (*p* = 0.043) better assessed the improvement of symptoms compared to the placebo group (Figure 3), and a statistically significant improvement between visits II and III was found only in the synbiotic group. Assuming that an improvement is present when patients rated their symptoms >4 points on the IBS-GIS scale, a rating of 4 means lack of improvement and <4 indicates worsening; it was shown that the percentage of patients assessing symptoms >4 points is higher in the synbiotic group compared to the placebo group. However, these results did not achieve statistical significance (*p* = 0.07) (Table 5). The analyses of adequate relief (IBS-AR) did not show any statistically significant discrepancies between the studied groups and visits (Figure 3).

### 3.3. Study Secondary Endpoints

**Figure A1:**
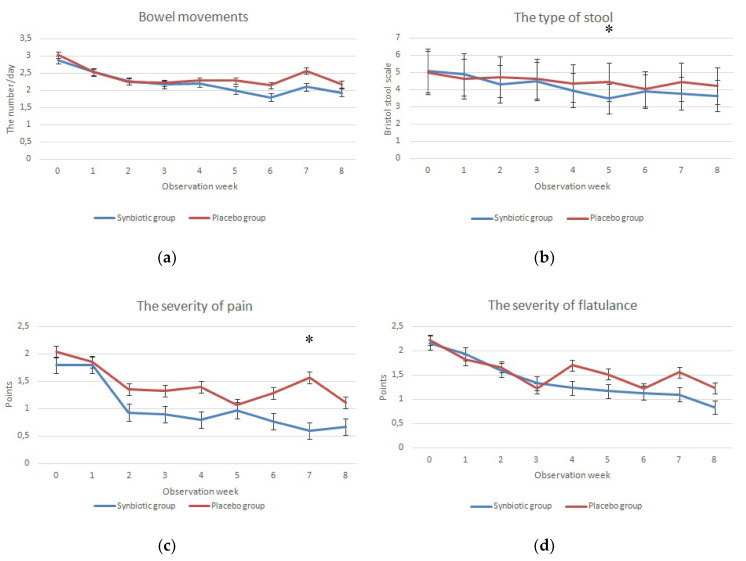

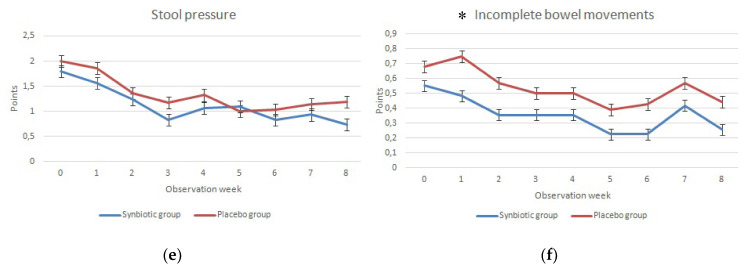
The impact of the synbiotic treatment on (**a**) bowel movements, (**b**) the type of stool, (**c**) the severity of pain, (**d**) the severity of flatulence, (**e**) stool pressure, (**f**) incomplete bowel movements assessed by the telephone interviewers. All data were collected during telephone interviews 3 times per week for 8 weeks. Week 0—screening week before enrollment into the study. Results are presented as a mean ± SD of the number of bowel movements per day, 5-point (for the severity of pain, flatulence, stool pressure) or 2-point (for incomplete bowel movements) scores and Bristol scale stool for the type of stool. * statistically significant (*p* < 0.05) differences between the synbiotic and placebo groups.

The secondary endpoints included the impact of the synbiotic intervention on the type of stools, the frequency of bowel movements per day, the severity of pain, flatulence and stool pressure, and the feeling of incomplete bowel movements. All data were collected by tele-interviewers. During 8 weeks of observation, a statistically significant improvement was observed in both groups for all IBS symptoms (data not shown, Figure A1). In patients treated with synbiotics, an improvement started earlier in terms of the frequency of bowel movements (in the 4th week of observation compared to the 5th week in the placebo group) and the type of stool (in the 2nd week compared to 5th week in the placebo group). The improvement in other symptoms occurred in the same weeks: the severity of pain in the 2nd week and the severity of flatulence and pressure of stool in the 3rd week.

**Figure 4:**
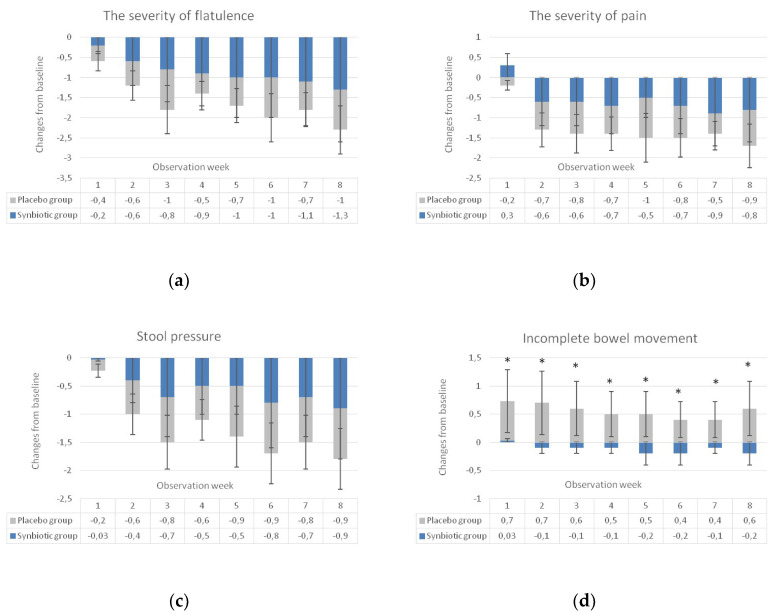
Weekly changes of IBS symptom scores of flatulence (**a**), abdominal pain (**b**), stool pressure (**c**), and a feeling of incomplete bowel movement (**d**) on a Likert scale (the lower the score the greater improvement). * Statistical significance between studied groups (*p* < 0.0001).

**Figure 5:**
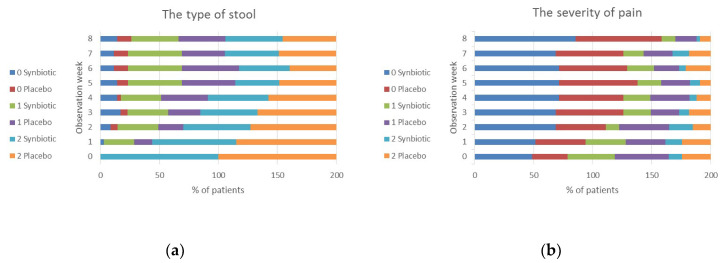

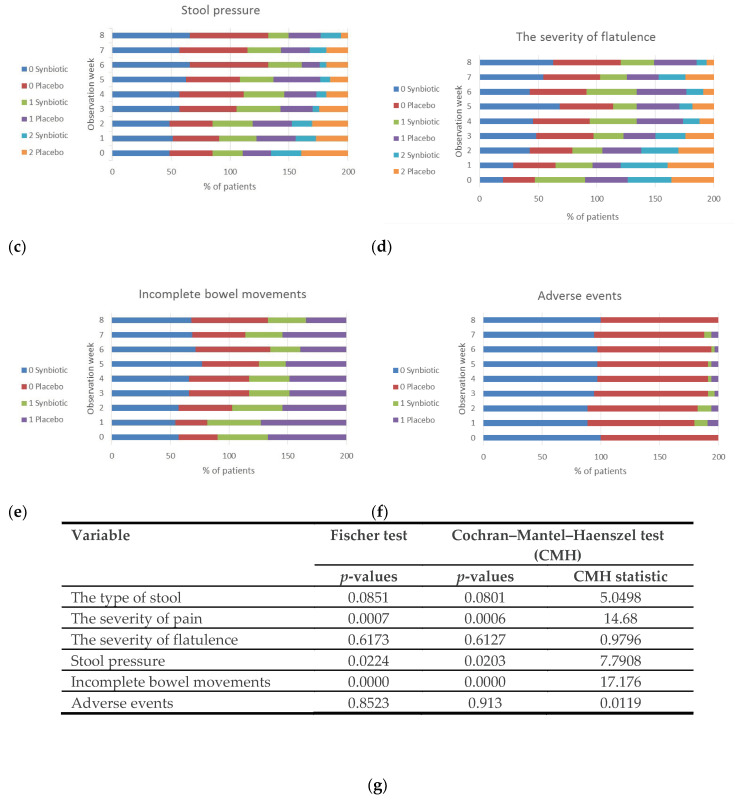
The impact of synbiotics on (**a**) the type of stool, (**b**) the severity of pain, (**c**) stool pressure, (**d**) the severity of flatulence, (**e**) incomplete bowel movements, (**f**) adverse events assessed by telephone interviewers. All data were collected during telephone interviews. Week 0—screening week before enrollment into the study (baseline). Results are presented as a percentage of patients who reported the severity of symptoms accordingly transformed into new scales described in Table 2. The table (**g**) presents *p*-values of a Fisher exact test and Cochran–Mantel–Haenszel test (CHM) and CHM statistic. 0 Synbiotic/0 Placebo—constipation, no or weak pain, flatulence and stool pressure, no feeling of incomplete bowel movements, no adverse events in the synbiotic/placebo group; 1 Synbiotic/1 Placebo—normal stool, intermediate pain, flatulence and stool pressure, presence of a feeling of incomplete bowel movements, presence of adverse events in the synbiotic/placebo group; 2 Synbiotic/2 Placebo—diarrhea, severe pain, flatulence and stool pressure in the synbiotic/placebo group.

When weekly changes of the scores from the baseline assessed in a 5-point Likert scale were analyzed (the lower scores were related to an greater improvement of symptoms) statistical differences (*p* < 0.0001) between studied groups were observed only for a feeling of incomplete bowel movements starting from the first week of observation and lasted for the entire 8 week observation period (Figure 4). The differences between the studied groups were also found when the scores of the analyzed symptoms were transformed according to the scheme presented in Table 2. A naïve statistical approach (a Fisher exact test), ignoring the fact that data for each patient were collected for nine weeks, showed that abdominal pain and stool pressure were significantly weaker in the synbiotic group compared to the placebo group. What is more, a feeling of incomplete defecation was significantly less common after the synbiotic treatment in comparison to the placebo (Figure 5). These results were confirmed with the use of the Cochran–Mantel–Haenszel test that accounts for the fact that data were collected for nine subsequent weeks.

A significant amelioration after the synbiotic treatment in comparison to placebo was also observed when nominal values presented as arithmetical means ± SD (Figure A1). A statistically significant improvement in the synbiotic group was observed for the stool type (evaluated in the Bristol stool scale), the severity of pain and the feeling of incomplete bowel movements, respectively in the 5th, 7th and 2nd week of observation.

### 3.4. Safety and Adverse Events

The synbiotic preparation was well tolerated. There were no statistically significant differences in adverse events between studied groups (Figure 5). At the beginning of therapy, 4 patients from the synbiotic group and 3 from the placebo group reported nausea, headache and rash (one patient from the placebo group). Reported symptoms resolved at week 8 in both groups.

## 4. Discussion

Synbiotics are potentially a promising approach in the treatment of functional bowel disorders, including IBS [30]. Synbiotics refer to the combination of synergistically acting probiotics and prebiotics, which are supposed to selectively stimulate growth and/or activate the metabolism of intestinal microbiota, thus having a beneficial effect on the host’s health.

This current randomized, double-blind, placebo-controlled study involving adult IBS patients indicates that the synbiotic preparation containing probiotic *Lactobacillus* (*Lactobacillus rhamnosus* FloraActive™ 19070-2, *Lactobacillus acidophilus* DSMZ 32418), *Bifidobacterium* (*Bifidobacterium lactis* DSMZ 32269, *Bifidobacterium longum* DSMZ 32946, *Bifidobacterium bifidum* DSMZ 32403) strains and scFOS has beneficial effects in the treatment of IBS-D. In our study, the multi-strain synbiotic therapy in comparison to the placebo significantly reduced the severity of IBS symptoms evaluated with both IBS-GIS and the total IBS-SSS scales. Furthermore, patients treated with synbiotics also reported in weekly observations a significant amelioration of individual IBS symptoms such as pain, flatulence, stool pressure, a feeling of incomplete bowel movements and the diarrheal type of stool compared to those receiving placebo.

The symptom severity score (IBS-SSS) in our IBS patients was in the range of moderate to severe and was similar in both studied groups prior to treatment. IBS symptoms significantly improved after 4 and 8 weeks of the intervention in both groups, but the significant difference in the total IBS-SSS score between the synbiotic group and the placebo group was achieved in the 8th week. Note that the placebo effect was very strong, especially after 4 weeks of treatment, and this could explain no significant effect of synbiotics during this treatment period as well as the lack of continuous significant differences in the assessment of individual IBS symptoms in weekly observation between the groups. The placebo effect could be enhanced by frequent telephone calls. Regular telephone inquiries from tele-interviewers could have been a form of psychological support for IBS patients.

Previous studies on the efficacy of multi-strain synbiotics in IBS management have provided different results. Shavakhi et al. [31] evaluated the impact of a synbiotic preparation containing *Lactobacillus* (*Lactobacillus casei*, *Lactobacillus rhamnosus*, *Lactobacillus acidophilus*, *Lactobacillus delbrueckii* ssp. *bulgaricus*), *Bifidobacterium* (*Bifidobacterium breve*, *Bifidobacterium longum*) and prebiotic FOS on the severity of abdominal pain and the distention in Iranian IBS patients (*n* = 126). Despite the observed improvement of symptoms in both study groups, no statistically significant differences were found between the synbiotic group and the placebo group. However, the observation time in this study was fairly short, only 2 weeks after administration of the synbiotic preparation, and the effects of synbiotics were limited to assessing only two symptoms. In our study, differences between the groups when we assessed the impact of synbiotics on pain only appeared in the 7th week of intervention, although a statistically significant reduction in severity of abdominal pain compared to the baseline in both studied groups occurred already in the 2nd week of the intervention. The earliest statistically significant differences between the studied groups, already present in the first week of observation, pertained to the feeling of incomplete bowel movement, but this symptom was not assessed by Shavakhi et al. [31]

Other randomized study of a synbiotic multi-strain preparation with inulin (*n* = 64) [32] showed a beneficial effect in decreasing the severity of flatulence in IBS patients but failed to achieve an improvement in global satisfactory relief of abdominal flatulence and bloating. Weekly evaluation of the severity of pain and urgency did not show any significant differences between the two groups during the 4-week lasting treatment period, and the longer observation was not performed.

In contrast to described randomized clinical trials evaluating multi-species synbiotics, Rough et al. analyzed the effect of a synbiotic preparation composed of a single species and FOS. Adult IBS patients (*n* = 85) were randomized to receive a synbiotic containing *Bacillus coagulans* or placebo for 12 weeks [33]. Frequency of IBS symptoms including abdominal pain, diarrhea and constipation was evaluated before and after the intervention and then after nine months follow-up. Authors observed significant reduction in frequency of abdominal pain and diarrhea in the synbiotic group compared with placebo and no effects on constipation. Although this synbiotic preparation had beneficial effects on selected IBS symptoms that were maintained at 9 months follow-up, many patients receiving synbiotics reported adverse events such as vomiting and diarrhea, which caused 41% dropout from the synbiotic group (compared with 25% dropout in the placebo group). In this aspect, these results differed from the findings of the current study and other clinical trials in which both probiotics and synbiotics were not associated with a higher risk of such adverse events and high dropout rates [17,31,32]. The synbiotic preparation used in our study was well tolerated, and occasional adverse events that resolved during the last week of therapy were observed in both treatment groups.

It is still an open question whether synbiotics as a mixture of probiotics and prebiotics are superior in IBS than probiotics alone [30]. The beneficial effects of probiotics were presented in many clinical trials and meta-analyses [17,18,19,20]. The mechanism of probiotic efficacy is supposed to be connected with the impact on the gut microbiota and its metabolome and with a direct activation of the gut associated lymphoid tissue as well as a modification of the microbiome-gut-brain axis. However, prebiotic oligosaccharides also play an important role in modulation of the intestinal microbiota and are the main source of short chain fatty acids such as acetate, propionate and butyrate [34]. Recent analyses of the gut microbiota of IBS patients have shown that microbial dysbiosis in IBS depends on IBS subtypes, and IBS-D but not IBS-C is characterized by a reduction of butyrate producing bacteria such as *Ruminococcaeae*, unknown *Clostridiales* and *Erisipelotrichaceae* [14]. As butyric acid is known to improve the intestinal epithelial barrier, this reduction could be responsible for an increase in the epithelial barrier permeability, which has been previously associated with IBS-D [35].

In our study, scFOS were used as a prebiotic component. scFOS are a group of linear fructose oligomers with a degree of polymerization ranging from one up to five (oligosaccharides), which are fully fermented by the colonic microbiota. In humans, increasing doses of scFOS supplementation between 2.5 and 10 g/day was associated with an increase in fecal bifidobacteria [36], and in an experimental piglet model, dietary FOS was responsible for an increase in butyrate production in the large intestine [37].

In a previous study on the effect of scFOS on digestive symptoms in subjects with minor functional bowel disorders, scFOS reduced the frequency of abdominal distension after 4 weeks of supplementation. This was not observed with placebo, even if the placebo effect was very strong (more than 70% of subjects felt improved intestinal discomfort) [38]. When scFOS were given alone to IBS patients in daily doses of 5 g, these prebiotic oligosaccharides significantly reduced anxiety scores compared with the placebo. Their use resulted in an increase in fecal bifidobacteria without modifying other bacterial groups [39]. In our study, less than 2 g of scFOS were given in daily dose; however, prebiotic oligosaccharides were given together with a 10 billion-dose of multi-species probiotic, and a synergistic effect of both components was to be expected. Studies comparing the effects of synbiotics with prebiotic and probiotic bacteria alone are rare. Such study was carried only in the group of pediatric patients with IBS [40]. Children received a synbiotic preparation containing 5 × 10^9^ CFU of *Bifidobacterium lactis* B94 and 900 mg inulin, probiotic alone (5 × 10^9^ CFU *Bifidobacterium lactis* B94), or prebiotic (900 mg inulin) twice daily for 4 weeks. This study presented superior effects of synbiotics on full recovery from IBS symptoms. In contrast, the addition of dietary fibers in the form of polydextrose to sterilized probiotic *Lactobacillus helveticus* did not appear to result in better health benefits in adult patients with IBS-C than the sterilized probiotics alone, whereas probiotics improved constipation-related syndromes [41]. However, this intervention did not contain live probiotic bacteria but heat inactivated strains.

### Limitations and Strengths of the Study

A trial of this type has a number of important limitations that need to be outlined. Firstly, a small number of patients was included in this study (80 patients, 68 of whom completed the study). Thus, the statistical power to determine statistically relevant differences between the synbiotic and placebo patients could be limited. Secondly, while all participants were advised to maintain their usual dietary practices throughout the study, which was monitored informally at visits, no nutritional assessments were undertaken to confirm dietary adherence. Thirdly, the duration of the study was limited to 8 weeks of therapy, and there was no follow-up observation. Although such scheme is consistent with clinical trials in this therapeutic setting, it is a relatively short period for a potentially life-long disease. Consequently, no conclusions regarding the durability of the response can be drawn. Finally, the placebo group obtained maltodextrin, which was not included in the synbiotic preparation, and it is uncertain whether some positive effects in the placebo group were due to this ingredient.

The strengths of this clinical trial relate to its design involving a relatively homogeneous group of patients with a defined subtype and severity (IBS-D with IBD-SSS score >175 points) and with strict controls to reduce factors which could influence bias (maintained double-blind by independent investigators). The vast majority of IBS probiotic/synbiotic trials undertaken so far are planned in such a way that the physician’s assessment takes place in intervals (usually several weeks between visits) and possibly keeping a diary with the assessment of symptoms by patients between scheduled visits. The strength of our study was the systematic contact with the patient three times a week by the same tele-interviewer, which allowed a relatively restrictive patient monitoring and adherence to the treatment. Another strong points of the study, such as continuous assessment of the severity of symptoms, registration of adverse events and the reasons for patients withdrawing from the study also should be highlighted.

## 5. Conclusions

The current randomized, double blind, placebo-controlled study involving adult IBS patients indicates that the synbiotic preparation containing probiotic *Lactobacillus rhamnosus* FloraActive™ 19070-2, *Lactobacillus acidophilus* DSMZ 32418, *Bifidobacterium lactis* DSMZ 32269, *Bifidobacterium longum* DSMZ 32946, *Bifidobacterium bifidum* DSMZ 32,403 strains and scFOS, in a dose of 5 billion of CFU and 947 mg twice daily has beneficial effects in the treatment of IBS-D. The synbiotic intervention was safe and induced an improvement in total IBS-SSS score, in IBS-SSS scores related to flatulence and bowel habit as well as in global IBS symptoms after 8 weeks of therapy as well as a decrease in the severity of IBS related symptoms such as a feeling of incomplete bowel movements, flatulence, abdominal pain, stool pressure and normalization of diarrheal stools compared with placebo in weekly observations. Thus, these results suggest that synbiotics offer a benefit for IBS-D patients. Further trials with longer duration of treatment and follow-ups will answer the question of whether an 8-week treatment is sufficient to maintain positive effects.

## Figures and Tables

**Table 1:** Probiotic composition of a synbiotic preparation used in a clinical trial.

Strain	Colony-Forming Units (CFU) Per Sachet	CFU Per Daily Dose (2 Sachets)	Strain Number
*Bifidobacterium lactis*	2.94 × 10^9^	5.88 × 10^9^	DSMZ 32269
*Bifidobacterium longum*	2.94 × 10^8^	5.88 × 10^8^	DSMZ 32946
*Bifidobacterium bifidum*	2.94 × 10^8^	5.88 × 10^8^	DSMZ 32403
*Lactobacillus rhamnosus*	9.80 × 10^8^	1.96 × 10^9^	FloraActive 19070-2
*Lactobacillus acidophilus*	4.90 × 10^8^	9.80 × 10^8^	DSMZ 32418
Total CFU	5.00 × 10^9^	1.00 × 10^10^	

**Table 2:** Scores assessing the type of stool and the severity of irritable bowel syndrome (IBS) symptoms based on the information obtained by telephone interviewers after transformation for statistical analyses.

Variable	Primary Score	Transformed Score
The type of stool	Bristol stool scale (points 1–7)	0—constipation if primary scale = 1 or 2 1—normal stool if primary scale = 3 or 4 2—diarrhea if primary scale = 5 or 6 or 7
The severity of pain, flatulence, stool pressure	5-point Likert scale	0—no or weak if primary scale = 0 or 1 1—intermediate if primary scale = 2 2—strong or very strong if primary scale = 3 or 4

**Table 3:** Patient characteristics.

	Synbiotic Group (*n* = 35) *n* (%) or Mean ± SD	Placebo Group (*n* = 33) *n* (%) or Mean ± SD
Gender		
Female	25 (71.4%)	24 (72.7%)
Male	10 (28.6%)	9 (27.3%)
Age in years	43.2 ± 14.0	36.7 ± 12.7
Weight in kg	74.9 ± 14.6	69.0 ± 14.2
Height in m	1.69 ± 0.08	1.7 ± 0.09
BMI	26.21 ± 5.43	23.86 4.65
IBS severity *		
Moderate	14 (40.0%)	10 (30.3%)
Severe	21 (60.0%)	23 (69.7%)
Total IBS-SSS score	318.1 ± 63.6	325.5 ± 49.9

* IBS severity was assessed with the use of IBS symptom severity scale (IBS-SSS). There were no significant statistical differences between patients receiving a synbiotic preparation (the synbiotic group) and placebo (the placebo group). BMI = Body Mass Index.

**Table 4:** The effect of synbiotic intervention on the severity of IBS symptoms assessed with the use of IBS-SSS score.

Groups	Baseline (Visit I)	Week 4 (Visit II)					Week 8 (Visit III)			
	Mean ± SD	Mean ± SD	Change from Baseline	*p*-Values within-Group	*p*-Values Comparison with Placebo		Mean ± SD	Change form Baseline	*p*-Values within-Group	*p*-Values Comparison with Placebo
Total IBS-SSS										
Synbiotic	318.1 ± 63.6	213.8 ± 58.7	−104.3 ± 88.2	<0.01		0.248	148.5 ± 51.2	−169.6 ± 88.7	<0.01	0.042
Placebo	325.5 ± 49.9	233.8 ± 81.7	−91.7 ± 82.0	<0.01		NA	183.7 ± 85.7	−141.8 ± 95.4	<0.01	NA
IBS-SSS 1 (the severity of pain)										
Synbiotic	55.7 ± 23.4	37.1 ± 21.3	−18.7 ± 28.8	<0.01	0.878		24.9 ± 12.2	−30.9 ± 24.7	<0.01	0.118
Placebo	60.6 ± 20.8	37.9 ± 17.8	−22.7 ± 29.5	<0.01	NA		31.8 ± 22.8	−28.8 ± 32.5	<0.01	NA
IBS-SSS 2 (the frequency of pain)										
Synbiotic	32.1 ± 18.5	20.3 ± 17.0	−13.0 ± 28.1	<0.01	0.509		12.5 ± 16.3	−19.6 ± 20.4	<0.01	0.151
Placebo	34.8 ± 21.6	23.5 ± 21.6	−11.4 ± 26.6	<0.01	NA		19.7 ± 24.0	−15.2 ± 29.9	<0.01	NA
IBS-SSS 3 (the severity of flatulence)										
Synbiotic	63.2 ± 30.2	42.4 ± 25.0	−20.8 ± 32.8	<0.01	0.039		23.6 ± 19.1	−40.1 ± 35.4	<0.01	0.028
Placebo	68.2 ± 18.0	55.3 ± 25.6	−12.9 ± 26.6	<0.01	NA		34.1 ± 19.6	−33.9 ± 29.9	<0.01	NA
IBS-SSS 4 (dissatisfaction with bowel habit)										
Synbiotic	83.5 ± 19.0	58.5 ± 16.3	−25.0 ± 29.7	<0.01	0.590		44.1 ± 21.3	−39.4 ± 30.0	<0.01	0.135
Placebo	80.2 ± 17.1	61.1 ± 22.6	−19.4 ± 22.0	<0.01	NA		52.1 ± 22.0	−28.4 ± 25.2	<0.01	NA
IBS-SSS 5 (quality of life)										
Synbiotic	83.5 ± 19.0	56.6 ± 17.2	−26.9 ± 22.7	<0.01	0.913		43.4 ± 21.0	−40.1 ± 25.4	<0.01	0.618
Placebo	81.5 ± 17.2	56.1 ± 21.2	−25.4 ± 22.3	<0.01	NA		46.0 ± 21.8	−35.4 ± 25.0	<0.01	NA

The severity of IBS symptoms was assessed with the use of IBS-SSS score before treatment (visit I), after 4 weeks (visit II) and 8 weeks (visit III) of the treatment. A score reduction was related to symptom amelioration. The results are presented as a mean ± SD and a change in IBS-SSS score from baseline.

**Table 5:** The effect of synbiotic treatment on the global improvement of IBS symptoms.

	Probiotic Group (*n* = 35)		Placebo Group (*n* = 33)		*p*-Value	OR
	Improvement	No Improvement	Improvement	No Improvement		
Visit II	27 (77.1)	8 (22.9)	24 (72.7)	9 (27.3)	0.7819	0.7929
Visit III	33 (94.3)	2 (5.7)	26 (78.8)	7 (21.2)	0.0794	0.2299

The global improvement of symptoms was assessed using IBS-GIS questionnaire after 4 weeks (visit II) and 8 weeks (visit III) of intervention. IBS-GIS score was recoded to indicate improvement (if IBS-GIS was > 4 points) or no improvement or worsening (if IBS-GIS was ≤ 4 points). The results are presented as number of patients and percentages (in brackets). OR = odds ratio.

## Appendix A

**Table A1:** The effect of synbiotic treatment on the improvement of IBS severity assessed with the use of IBS-SSS score.

	Synbiotic Group (*n* = 35)		Placebo Group (*n* = 33)		*p*-Value	OR
	Improvement	No Improvement	Improvement	No Improvement		
IBS-SSS 1 (the severity of pain)						
Visit II	6 (17.1)	29 (82.9)	9 (27.3)	24 (72.7)	0.387	1.797
Visit III	13 (37.1)	22 (62.9)	10 (30.3)	23 (69.7)	0.614	0.739
IBS-SSS 2 (the frequency of pain)						
Visit II	6 (17.1)	29 (82.9)	5 (15.1)	28 (84.9)	1.000	0.865
Visit III	6 (17.1)	29 (82.9)	6 (18.2)	27 (81.8)	0.749	1.328
IBS-SSS 3 (the severity of flatulence)						
Visit II	12 (34.3)	23 (65.7)	8 (24.2)	25 (75.8)	0.431	0.618
Visit III	18 (51.4)	17 (48.6)	12 (36.4)	21 (63.6)	0.232	0.545
IBS-SSS 4 (dissatisfaction with bowel habit)						
Visit II	8 (22.9)	27(77.1)	1 (3.0)	32 (97.0)	0.028	0.108
Visit III	14 (40.0)	21 (60.0)	7 (21.2)	26(78.8)	0.119	0.409
IBS-SSS 5 (quality of life)						
Visit II	5 (14.3)	30 (85.7)	14 (40.0)	21 (60.0)	0.710	0.604
Visit III	3 (9.1)	30 (90.9)	10 (30.3)	23 (69.7)	0.454	0.656

The results are presented as a number and percentage (in brackets) of patients reported an improvement/no improvement of symptoms assessed by IBS-SSS scale after synbiotic intervention lasting 4 weeks (visit II) and 8-weeks (visit III). An improvement was defined as a 50-point decrease compared to baseline.
